# Mechanical and Strain-Sensing Responses in Functional ZnO Tetrapod - Silk Fibroin Composites: A Detailed Investigation of the Roles of Filler Size and Shape

**DOI:** 10.1021/acsomega.5c13118

**Published:** 2026-06-18

**Authors:** Rocco Malaspina, Martina Alunni Cardinali, Hamed Haftbaradaran, Danila Maltsev, Hira Abdullah, Horst-Günter Rubahn, Alessandro Di Michele, Anna Donnadio, Paola Sassi, Yogendra Kumar Mishra, Nicola M. Pugno, Luca Valentini

**Affiliations:** † Department of Physics and Geology, 225037 University of Perugia, Via A. Pascoli, Perugia 06123, Italy; ‡ Department of Chemistry, Biology and Biotechnology, 9309 University of Perugia, Perugia 06123, Italy; § Mechano-X Labs, Department of Civil, Environmental, and Mechanical Engineering, 217740 University of Trento, Trento 38123, Italy; ∥ Department of Civil and Environmental Engineering, University of Perugia, Via G. Duranti, Perugia 06125, Italy; ⊥ Smart Materials, Mads Clausen Institute, 362638 University of Southern Denmark, Alsion, 2, Sønderborg DK-6400, Denmark; # Department of Pharmaceutical Science, University of Perugia, via Del Liceo 1, Perugia 06123, Italy; ∇ School of Engineering and Materials Science, Queen Mary University of London, London E1 4NS, U.K.

## Abstract

In this study, functional composites were developed by dispersing ZnO tetrapods (ZnO-T) in a solution of silk fibroin (SF) and formic acid (FA). Micro- and nanoparticles with different shapes were observed in the SF matrix after evaporation of the solvent, depending on ZnO-T filler content. The secondary structure of SF, as well as the shape (e.g., spherical, linear, or branched) and aspect ratio of the fillers, were investigated by FTIR spectroscopy, optical and scanning electron microscopy. While FTIR spectroscopy indicates that the secondary structure of SF is not affected by the size and shape of ZnO-T, the observed variation in effective elastic modulus measured for composites is correlated with different filler particle aspect ratios, predicting the formation of a stiff interface. We observed that branched tetrapodal fillers exhibit significant potential for optimization of composites’ mechanical properties in comparison to their spherical and linear counterparts. By combining ZnO microparticles with SF, we fabricated a stretchable strain sensor that demonstrated an appreciable improvement in the fractional change of electrical resistivity and gauge factor with respect to neat SF films.

## Graphical abstract

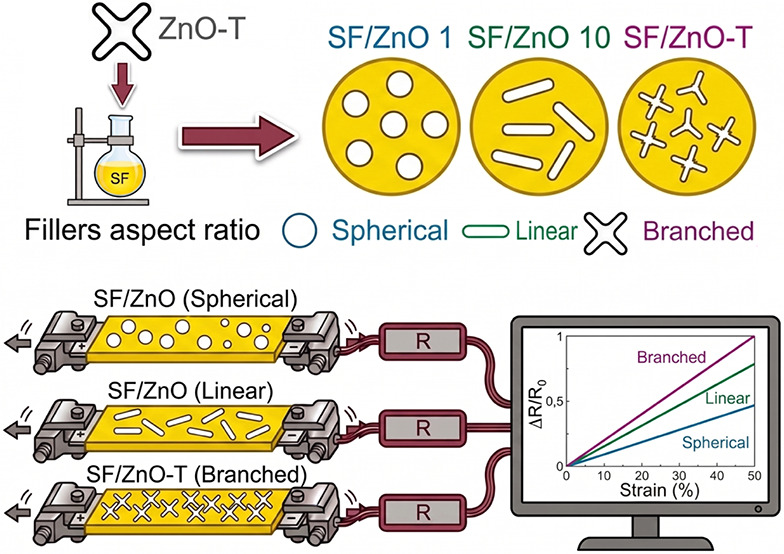

## 1 Introduction

Silk fibroin, a natural biopolymer, has been extensively investigated due to its biocompatibility, biodegradability, and mechanical properties.^1^ These characteristics make it an attractive candidate for various applications, including the development of advanced composites. As an example, in a previous work, we showed that a hybrid SF/black phosphorus film can significantly reduce the proliferation of *Candida albicans* cells.^2^ Moreover, silk-based composites have shown the potential for applications in wound healing,^3^ bone regeneration,^4^ and pressure sensing.^5^

However, to optimize its performance, particularly in composite materials, the regeneration of the crystalline β-sheet conformation in SF is essential. Indeed, this process is key to enhancing the hydrophobicity, thermal stability, mechanical strength, and antibacterial properties of the material^6–8^ .

It is well-known that the properties of polymers can be improved by the incorporation of different types of rigid particles.^9^ Improvements in mechanical performance in terms of stress–strain behavior depend on the shape (e.g., aspect ratio) of filler particulates. The utilization of carbon nanotubes (CNTs), for example, has demonstrated that a high aspect ratio of fillers contributes to the reinforcement of the silk-based composite.^10,11^ Introduction of inorganic filler particles induces a nucleating effect in SF nanocomposites with improved performance in elongation at break and toughness.^8^ A large variety of inorganic nanostructures have been used as fillers in SF matrices.^12,13^ It has been observed that the nanostructure morphology, surface texture, and surface chemistry play a significant role in the nanocomposites’ overall response.^14,15^ While hybrid-material SF films present a broad field of research, state-of-the-art studies rarely involve complex 3D morphologies of the fillers. For example, to the best of our knowledge, there are no reported studies of hybrid composites of SF with tetrapodal-shaped dopants made of ZnO or other compounds. Conventional zero-, one-, and two-dimensional particles suffer from several limitations, such as agglomeration and uneven distribution, which hinder the desired response of the target materials. Complex 3D structures as dopants could play a prominent role in functional engineering, aiming for materials with a specific response.

Tetrapodal crystals consist of four 1D rod-shaped arms that are interconnected via a central crystalline core. The arms form tetrahedral angles of approximately 105°–110° with respect to each other.^16–19^ The tetrapodal shape of micro- and nanostructures offers a high aspect ratio in three dimensions, enabling its advanced applications in polymer composite research, such as improving tensile strength, fracture toughness, as well as electrical and dielectric properties. ZnO-T represents a promising candidate for applications in flexible electronics and sensing.^20^ For instance, it has been demonstrated that a ZnO-T-based device can exploit bending-induced piezoelectric effect.^21^ In particular, Yin et al. showed by finite element simulations that the tetrapodal structure enhances the sensing properties of the composite, compared to typical ZnO hexagonal nanorods, due to the possibility of being used as multiterminal strain sensors.^22^ These piezoelectric properties of ZnO-T can be exploited in the context of pressure sensing too, as demonstrated by Pandit et al.^23^ Some polymer composites containing ZnO-T change their coloration in response to applied stress: Liao et al.^24^ have demonstrated the utilization of ZnO for resistance-based strain sensing. The microtectonic sliding of oxide plates under strain was found to be the mechanism that caused the resistance change. This sensing mechanism is very common in oxide-based thin films, including ZnO.^25^

A composite film of SF and ZnO nanoparticles was developed via spin-coating, utilizing piezoelectric properties. The device generated up to 25 V and 6.67 mW/cm^3^, successfully powering red LEDs.^26^ The inclusion of ZnO nanoparticles resulted in typical n-type behavior, providing semiconductor functionality and high optical transmittance.^27^ Using biomineralization, flower-like ZnO nanostructures were deposited onto SF filaments and studied for red photoluminescence, with potential medical and antibacterial applications.^27^ The ZnO nanostructures deposited onto fibroin hydrogels enhanced mechanical properties and water retention (up to 2500%), making them suitable for biomedical dressings. The SF-ZnO coatings on TiO_2_ nanotubes showed antibacterial activity (greater than 50%) and improved stability and electrochemical capacity. SF-ZnO scaffolds (with cerium doping) were studied for bone tissue engineering, showing appropriate porosity, controlled degradation, biocompatibility, and support for cell proliferation.^28^ A comprehensive review analyzed SF-based scaffolds incorporating ZnO (alongside FeO, CuO, and MgO) nanostructures, highlighting their role in healing devices and mechanical enhancement.^29^ To the best of our awareness, there exist no such studies related to ZnO-T and SF composites: this could be a crucial development for a very broad scientific community.

In this work, silk fibroin (SF)-based composite films incorporating microscale ZnO-T particles were fabricated and systematically characterized, with particular emphasis on their mechanical behavior and filler dispersion within the matrix. The influence of filler morphology was investigated by comparing three types of ZnO inclusions: (i) globular microparticles, (ii) rod-like structures, and (iii) branched tetrapodal (ZnO-T) microcrystals. This comparative analysis provides insight into the role of particle geometry on dispersion quality, interfacial interactions, and stress transfer efficiency within the polymer matrix. The results highlight the relevance of filler architecture in governing load-bearing mechanisms and the overall mechanical performance of the composites. A preliminary assessment of the piezoresistive response is also presented, mainly to support the interpretation of underlying structure–property relationships, while potential functional implications are briefly outlined.

## 2 Materials

The microscale ZnO-T has been produced by a simple flame transport synthesis (FTS) approach.^16,17,20,30^ In the FTS process, Zn microparticles are mixed with polyvinyl butyral powder in a 1:2 weight ratio, which serves as the main precursor material. The precursor mixture (in a ceramic crucible) is heated in a muffle furnace at 900 °C for 30 min. The conversion of Zn microparticles into microsized ZnO-T occurs inside the flame via a solid–vapor–solid growth mechanism. After the process, a white, snowflake-like powder of ZnO-T is collected from the furnace and used for desired applications. The FTS process is very simple and can be easily upscaled (several kilograms of ZnO-T have already been produced).

The silk cocoons were supplied by a local farm (Fimo S.r.l., Milano, Italy). Calcium chloride, formic acid (FA), and NaHCO_3_ were supplied by Sigma-Aldrich. Silk solutions were prepared as reported previously.^31,32^ Silk cocoons were degummed with NaHCO_3_ and dispersed into 5 mL of FA/CaCl_2_ solution, where CaCl_2_ weigh ratio to SF was 30/70, i.e., for 5 mL of FA, 700 mg of SF and 300 mg of the salt were combined. SF films were produced by dry casting onto Petri dishes overnight, with subsequent annealing at 40 °C for 2 h.

SF/ZnO films were fabricated by adding 0.1, 1, and 10 wt % of ZnO-T to the silk solutions, respectively, in an FA/CaCl_2_ solvent. We highlight that every operation performed with FA was carried out under a fume hood while wearing the necessary PPE (gloves, goggles, and a lab coat) to ensure operator safety.

To elucidate the role of FA on ZnO-T morphology, SF films were then redissolved in PBS 1× (pH 7.4) and sonicated for 2 h at room temperature. Then, 10 wt % of ZnO-T with respect to the SF mass was added to the SF solution. SF/ZnO-T films were produced as well by dry casting onto Petri dishes over 12 h. Since ZnO is highly reactive with FA, forming zinc formate, we produced different SF-ZnO films by dissolving 10 wt % of ZnO powder (with no tetrapodal morphology) in FA for the purpose of comparing the mechanical properties of SF films fabricated from a different ZnO powder. Henceforth, these samples are labeled SF/ZnO-P. The full nomenclature of the samples is summarized in Table 1 below.

**Table 1:** Definitions of Abbreviated Names Assigned to the Samples

Sample	Preparation
SF	Silk fibroin dissolved in FA/CaCl_2_
SF/PBS	Silk fibroin redissolved in PBS
SF/ZnO 0.1	Silk fibroin dissolved in FA/CaCl_2_ with 0.1 wt % of ZnO-T
SF/ZnO 1	Silk fibroin in FA/CaCl_2_ with 1 wt % of ZnO-T
SF/ZnO 10	Silk fibroin in FA/CaCl_2_ with 10 wt % of ZnO-T
SF/ZnO-T	Silk fibroin redissolved in PBS with 10 wt % of ZnO-T
SF/ZnO-P	Silk fibroin dissolved in FA/CaCl_2_ with 10 wt % of ZnO powder (nontetrapod morphology)

### 2.1 Materials Characterization

The microscopic images were obtained using a SUPRA 40 VP Field Emission Scanning Electron Microscope (FESEM) at 2 kV. Optical images of ZnO-T, as well as of ZnO particles after FA evaporation, were recorded with a 3D Hirox digital microscope (HRX-01) at 3000×, FOV (H)­104.04 μm, and resolution 0.04 μm. Optical microscopy images were then utilized to evaluate the aspect ratio of the filler. Globular-shaped filler particles were approximated with weakly eccentric ellipses, so the aspect ratio was calculated as the proportion between the long and short axes of the ellipse. Rod-shaped inclusions were approximated by cylinders; therefore, their aspect ratio was obtained as the proportion between the length of the rod (correspondingly, the height of the cylinder) and its diameter. For ZnO-T, the aspect ratio was taken to be that of its arms, and the calculation was performed in the same way as for rod-like fillers.

X-ray diffraction spectra were collected on the films with an AERIS Malvern diffractometer with a Cu target and Kα radiation (λ = 1.5418 Å) at 40 kV beam voltage and 40 mA current. The data were collected in the 5–80° 2θ range, with steps of 0.02°.

Infrared spectra were recorded on the films using a Fourier Transform Alpha spectrometer from Bruker Optics and its ATR (Attenuated Total Reflection) module equipped with a diamond crystal. At least three measurements were acquired on different regions of each film using a spectral range from 400 to 5000 cm^–1^ with a resolution of 2 cm^–1^ and an acquisition time of 128 scans. A background spectrum was acquired using the same number of scans before each measurement. Spectral data were preprocessed by tracing a straight baseline from 1740 to 1560 cm^–1^. Analysis of the secondary structure of silk fibroin of each sample, except for the SF/ZnO 10, was carried out by performing a curve fitting of the Amide I (1600–1700 cm^–1^) and Amide II (1500–1650 cm^–1^) bands with Gaussian profiles. Assignment of peaks in the Amide I region was performed according to the previous work of Lu et al.,^33^ while the Amide II band was fitted with a combination of 5 Gaussians.

The width of each spectral component was constrained to less than 30 cm^–1^, following what is reported in the literature.^34^ Calculation of the percentage for each secondary-structure component was carried out as the ratio between the area of the signals corresponding to that component and the total area of the Amide I band.

For the SF/ZnO 10 specimen, the procedure was slightly modified because the presence of zinc formate altered the region between the amide bands. To account for this, a prefit was performed using components obtained from the neat silk fibroin spectrum, fixed except for their amplitudes, along with an additional component representing the zinc formate peak. This was then subtracted from the spectrum, and the previously described fitting procedure was applied. The curve-fitting procedure was carried out using the Peak Analyzer tool of OriginLab software.

UV–vis reflectance measurements were performed with an Optosky ATP9310 microspectrophotometer equipped with a 20× objective. Before taking measurements, the signals of two reference surfaces (dark and white) were taken, and the reflectivity of the sample for a given wavelength was normalized from 0% to 100%, where 0% corresponds to the reflectivity of the dark reference and 95% to the reflectivity of the white one.

A custom-made testing device (AS Tessuti, Asper S.r.l., Italy) was employed to assess the elastic modulus and films’ resistance to tensile load. Each film was mounted using miniature clamps, and stress–strain curves were generated at a constant strain rate of 120% min^–1^ until failure. The tensile test film samples were die-cut to the dimensions of 20 mm working length and 5 mm width, with an average thickness of 370 μm. Each sample was tested a minimum of three times, with error bars representing the standard deviation.

To investigate the piezoresistive properties, tensile loads were applied to all specimens. A Kapton tape was placed between the loading platens and specimens to prevent direct contact. Electrical resistance data were recorded in DC mode with a Keithley 4200 SC synchronized with the tensile machine. This approach enabled reconstruction of plots that combined electrical resistance and engineered strain data, providing a unified view of the material response under strain.

Differential scanning calorimetry (DSC) was performed on a Mettler Toledo DSC-1 STARe system at 10 °C/min under a nitrogen flow of 50 mL/min over the 25–700 °C range. Data were processed using STARe software.

Thermogravimetric analyses (TGA) and derivatives of thermal gravimetry (DTG) were obtained by a TG–NETZSCH STA 2500 Regulus with a heating rate of 10 °C/min under an airflow of 50 mL/min.

### 2.2 Mechanical Model

In terms of the elastic moduli of the matrix and fillers, *E*_m_ and *E*_f_, and in the presence of an interphase with modulus *E*_int_, the effective modulus *E*_c_ of the composite containing randomly oriented fibers using the modified Halpin–Tsai theory^35^ has been estimated as

1 $$E_{c}=aE_{L}+\left(1-a\right)E_{T}$$

where

2 $$E_{L}=E_{m}\left(\frac{1+\xi \eta_{L,f}v_{f}+\xi \eta_{L,\mathrm{int}}v_{\mathrm{int}}}{1-\eta_{L,f}v_{f}-\eta_{L,\mathrm{int}}v_{\mathrm{int}}}\right)$$

3 $$E_{T}=E_{m}\left(\frac{1+2\eta_{T,f}v_{f}+2\eta_{T,\mathrm{int}}v_{\mathrm{int}}}{1-\eta_{T,f}v_{f}-\eta_{T,\mathrm{int}}v_{\mathrm{int}}}\right)$$

4 $$\eta_{L,f}=\frac{E_{f}-E_{m}}{E_{f}+\xi E_{m}}$$

5 $$\eta_{T,f}=\frac{E_{f}-E_{m}}{E_{f}+2E_{m}}$$

where *ξ* = 2*l*/*d* is the aspect ratio of the fibers, *E*_L_ and *E*_T_ represent the estimated moduli of the composite in the longitudinal and transverse directions of the fibers, and *η*_L,int_, *η*_T,int_ are found from eqs 4 and 5 with *E*_f_ replaced by *E*_int_. Here, *a* is a constant which sets the relative contribution of longitudinal and transverse directions to the overall load-carrying behavior, *v*_int_ is the volume fraction of the interphase, and *v*_f_ is the volume fraction of the fillers, here ZnO.^36^ The latter is related to the weight fractions *w*_f_ and *w*_m_ of the filler and matrix, respectively, through $v_{f}=\frac{w_{f}}{w_{f}+\frac{\rho_{f}w_{m}}{\rho_{m}}}$ , *ρ*_f_ = *ρ*_ZnO_ = 5.6 g/cm^3^, and *ρ*_m_ = 1.3g/cm3 ^37^ being the densities of ZnO and the SF matrix.

## 3 Results and Discussion

The ZnO-T consists of uniform hexagonal cylindrical arms with narrowing flat tips (Figure 1a and 1b). ZnO-T morphologies were analyzed by SEM, as shown in Figure 1c-e and Figure S1. ZnO-T has a diameter of about 4 μm at its base and ∼1 μm at the tips. ZnO-T arm length is in the range of 20–30 μm (Figure 1b). It can be observed that, along with the 3D geometry, each arm of ZnO-T exhibits superficial textured patterns, which function as active sites for chemical reactions. The surface of the ZnO structure is by default oxygen-deficient, leaving behind Zn^2+^ active polar sites on the surface. The 3D geometry, textured arm morphology, and Zn^2+–^terminated polar surfaces of ZnO-T set up a strong base for improved chemical reactivity during the composite formation.

**Figure 1:**
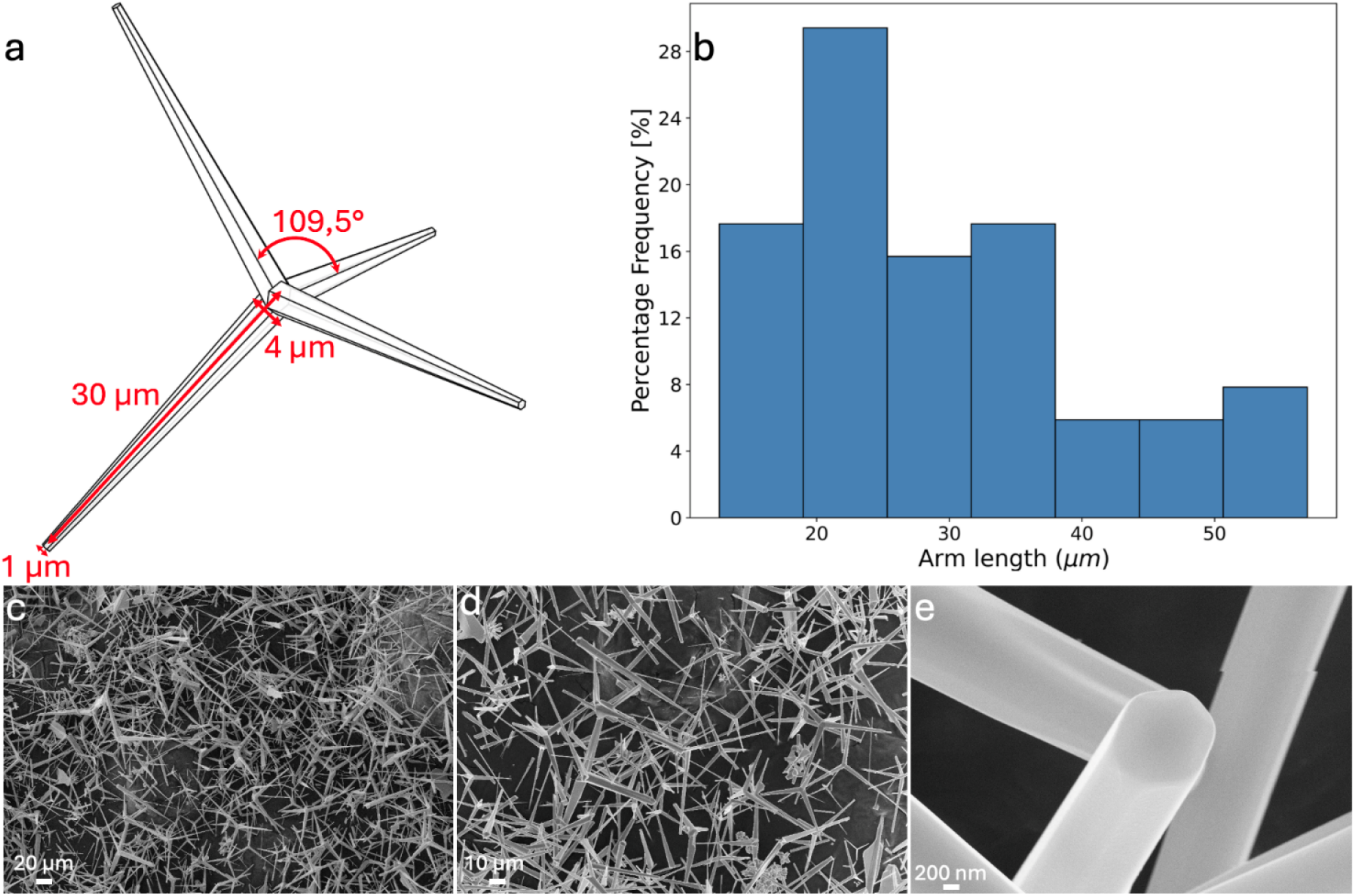
(a) Model showing the geometry of ZnO-T. (b) Histogram showing distribution of ZnO-T’s arm length. (c–e) FESEM images of ZnO-T material right after synthesis. (c) Network of ZnO-T, (d) magnified view of ZnO-T network, and (e) magnified FESEM image showing a hexagonal cylindrical tip of an arm.

The interaction of formic acid (HCOOH) with ZnO surfaces has been addressed in several studies,^38–41^ and several different possible formate structures have been proposed to result from dissociative adsorption of formic acid on the ZnO surface. It is also well known how complex microstructures can be fabricated through a self-assembly process.^42^ In such processes, the coalescence of initial ZnO nanoparticles in a liquid environment leads to different structures.^42,43^

Thus, we investigated the role played by ZnO-T concentration on the morphology of the particles after FA evaporation (Figure 2 and Figure S1). A typical optical image of ZnO-T filler is shown in Figure 2a. These ZnO-T exhibit four arms with an aspect ratio of 32.08 ± 14.78 for each arm. The globular particles were obtained by adding 1 wt % to FA and feature an aspect ratio of 1.53 ± 0.40, and form agglomerates (Figure 2b and 2e). While for tetrapodal particles there exists empty volume in between (for sterical reasons); globular ZnO particles are more closely packed. Microsized rod fillers have been obtained, with an aspect ratio of 10.27 ± 6.97 (Figure 2c and 2f), by increasing the ZnO-T content to 10 wt %. This morphology was maintained in the SF/ZnO 10 film as well (Figure 2d).

**Figure 2:**
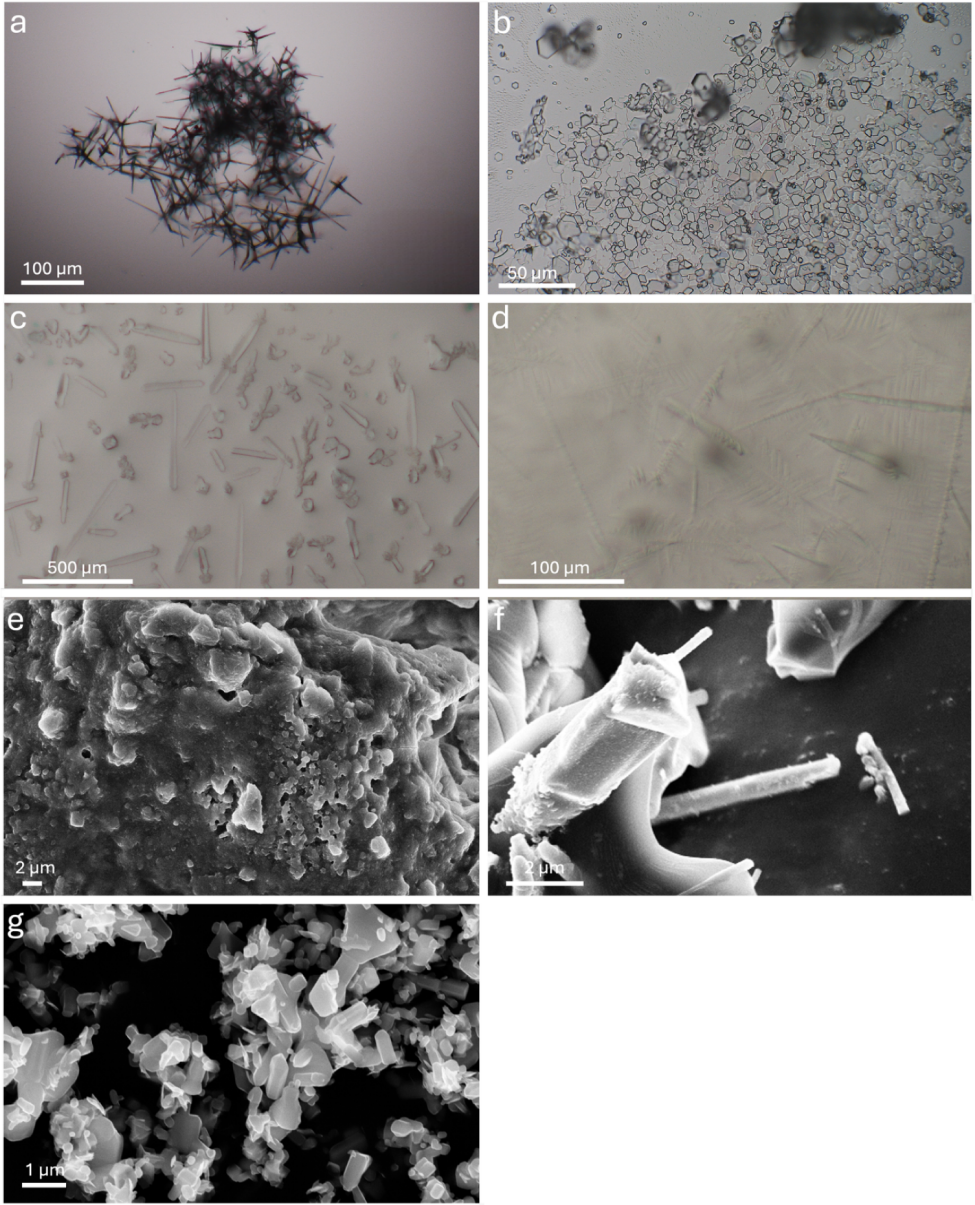
Optical images of (a) ZnO-T, (b) agglomerated globular particles, and (c) microrods obtained from FA solutions with different ZnO content (e.g., 1 and 10 wt %) after evaporation of the solvent. (d) Optical image of the SF/ZnO 10 film, where microrods dispersed in the SF matrix are well evident. FESEM images of (e) SF/ZnO 0.1, (f) SF/ZnO 10, and (g) SF/ZnO-P films, respectively.

ATR-FTIR spectroscopy was employed to analyze how SF secondary structure is affected by the interaction with different ZnO microstructures. Figure 3a and 3b present the spectral profiles of all SF/ZnO samples, which largely mirror those of pure SF, especially at lower oxide concentrations. However, noticeable changes appear in the SF/ZnO 1 and SF/ZnO 10 samples. In these, a progressive increase in intensity at 788, 1350, and 1572 cm^–1^—peaks characteristic of the formate species—confirms the reaction between FA and ZnO. In the high-frequency region (2700–3700 cm^–1^), a reduced intensity at 3400 cm^–1^ in the SF/ZnO 10 sample suggests smaller water content, indicating lower hygroscopicity.

**Figure 3:**
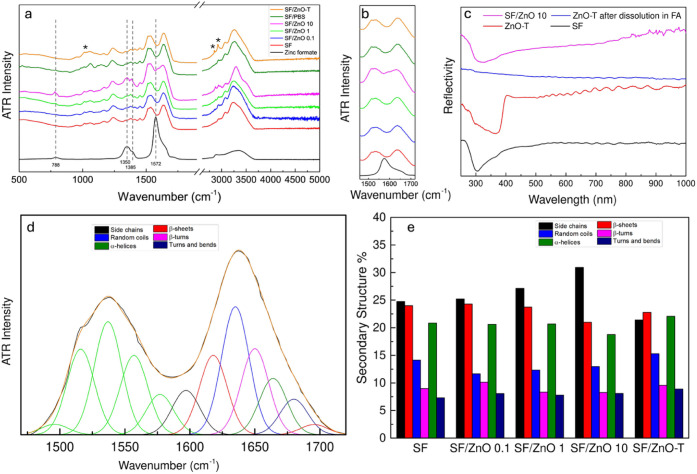
(a) ATR-FTIR spectra of zinc formate, SF, and SF/ZnO films; *: SF signals enhanced by interaction with ZnO (see text). (b) Zoomed-in view of the Amide bands of different samples. The coloring follows the same scheme as in the previous panel. (c) Comparison between UV–vis reflectance spectra of SF (black line), SF/ZnO 10 films (pink line), ZnO-T dry powder (red line), and ZnO-T dissolved in FA (blue line). (d) Example of curve fitting, with the sample being SF/ZnO 1. Experimental data are depicted with a black curve, reconstructed data after fitting are depicted in orange, and the various bands are depicted in other colors. (e) Relative weight of components obtained by curve-fitting procedure of ATR-FTIR spectra of SF and SF/ZnO films.

Optical properties of SF/ZnO films were also investigated using UV reflectance spectroscopy. Figure 3c shows the spectral profile of the SF/ZnO 10 sample, which exhibits a signal similar to that of pure SF at 307 nm, albeit broader and red-shifted. This shift is likely due to the contribution of ZnO, which typically shows a maximum absorption near 380 nm. These findings suggest that, although the presence of FA partially dissolves ZnO-T structures, most of the platelets or cylindrical units remain.

In contrast, ATR-FTIR spectra of SF/ZnO-T samples, shown in Figure 3a, do not show formate-characteristic peaks, indicating that ZnO-T remained unreacted, having avoided exposure to FA. Instead, the interaction between SF and ZnO-T is evidenced by increased intensity of SF signals at 1020, 2850, and 2920 cm^–1^. The 1020 cm^–1^ band is attributed to C–O stretching, while the bands at 2850 and 2920 cm^–1^ correspond to symmetric and antisymmetric CH_2_ stretching vibrations, respectively. Although differences in the amount of unreacted ZnO between the samples may contribute to the observed spectral variations, the enhanced intensity of these IR bands could arise from polarization of associated functional groups induced by their interaction with the ZnO surface. This effect might be further amplified at highly curved tips of tetrapod-shaped ZnO particles, where reduced dimensions can increase local electric fields, as previously reported in the literature.^44^ Nevertheless, verification of this hypothesis would require additional investigation, which is beyond the scope of the present work.

Amide I (1600–1700 cm^–1^) and Amide II (1500–1650 cm^–1^) FTIR bands (see Figure 3b) were used to monitor the conformational changes of the SF backbone.

By focusing on the Amide I region, we determined the percentages of different secondary structures (β-sheets, turns, and random coils) (see Figure 3d). As illustrated in Figure 3e, the presence of ZnO microparticles does not affect the contribution of β-sheet structure. Specifically, β-sheet content in all samples was found to remain at approximately 22%. Overall, the series of XRD spectra (Figure S2) demonstrates a systematic and linear-like increase in ZnO diffraction intensity with increasing tetrapod concentration, confirming homogeneous incorporation of the inorganic phase and the preservation of the intrinsic crystalline structure of ZnO within the composite films.

The change in the intensity proportion of three main reflections in the range of 2ϑ = 30–40° as well as newly emerged peaks between 2ϑ = 15–30° in the SF/ZnO-10 pattern (Figure S2) arises from a structural defect posed by formic acid. DSC and TGA thermograms (Figure S3) show, along with water trapped in the films, the formation of zinc formate, which is also in accordance with the FTIR results.

The tensile response of SF and SF/ZnO films at different percentages has been measured, and the results have been compared with those of SF/ZnO-T and SF/ZnO-P films (Figure 4a and 4b). The elastic modulus and mechanical strength of the composites increase with filler content. Figure 4b indicates that ZnO-T-reinforced SF composites exhibit the highest stiffness. The observed increase in the elastic modulus of SF, despite no change in the β-sheet fraction with higher filler content, is due to the replacement of SF volume with rigid filler domains. The observed differences are a physical effect induced by the three distinct particle shapes. ZnO-T has a higher aspect ratio than the other two types of fillers, so they require larger force to reorient in the direction of the external force, thus further reducing the deformability of the composite material. On the contrary, even if globular ZnO particles have a high surface-to-volume ratio and theoretically the highest degree of filler–polymer interaction, they exhibit the problem of agglomeration. Finally, SF/ZnO-P films show the highest elongation at break along with improved toughness.

**Figure 4:**
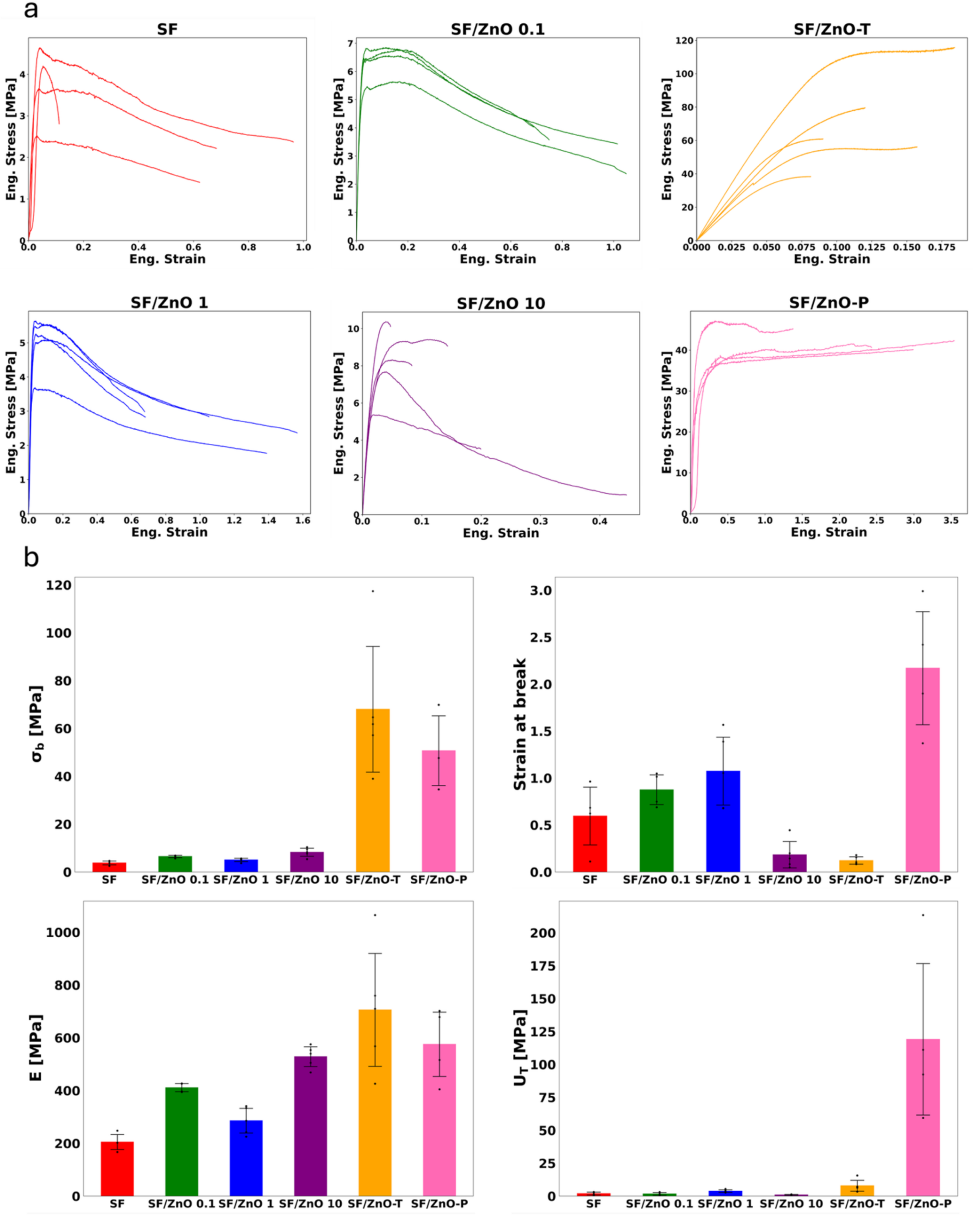
(a) Stress–strain curves of SF, SF/ZnO, SF/ZnO-T, and SF/ZnO-P films, respectively. (b) Strain at break, strength, toughness, and elastic modulus of the same set of samples.

It is of interest to examine the mechanical performance of the composites in terms of available composite theories, with the aim of understanding the load-bearing mechanisms involved.

To apply the theoretical framework to the experimental data, we first consider the SF/ZnO-P specimen, containing 10 wt % ZnO powder (not tetrapodal) and an average particle diameter *D* of ∼260 nm (Figure 2g). Assuming the absence of an interphase, and using the values of *E*_m_ = 204.86 MPa and *E*_f_ =130 GPa, together with particle aspect ratios ranging from 1 to 1.5, with *a* = 3/8, one obtains an estimate of *E*_c_ = 220.42 MPa. Note that the choice of *a* = 3/8 adopted based on previous studies^45,46^ is consistent with the random distribution of the fibers within the film-like geometry of the tested specimens. On the other hand, using *E*_f_ = 247.73 MPa, i.e., the maximum measured value for SF, the estimate increases to *E*_m_ = 267.01 MPa. Both estimates are significantly lower than the average modulus of *E* = 573 MPa found experimentally, hence suggesting that the formation of a stiff interphase is likely responsible for the discrepancy. Using *E*_int_ *≈* 10*E*_m_ = 2048.6 MPa for the interphase, numerical fitting using $\nu_{\mathrm{int}}\approx \nu_{f}\left[{\left(1+\frac{2t}{D}\right)}^{3}-1\right]$ and $\nu_{\mathrm{int}}\approx \nu_{f}\left[{\left(1+\frac{2t}{D}\right)}^{3}-1\right]$ , *t* being the interphase thickness,^36^ indicates that an interphase thickness of *t* ≈ 215 nm based on *E*_m_ = 204.86 MPa is required for the theoretical prediction to match the experiment. The required interphase thickness reduces to *t* ≈ 190 nm based on *E*_m_ = 247.73 MPa. While the estimated thicknesses are based on an order-of-magnitude increase in the interphase modulus compared to the SF matrix, i.e. for *E_int_=* 10*E*_m_, one may note that larger interphase thicknesses of *t* ≈ 368 nm and 248 nm are estimated based on the moduli ratio of *E*_int_/*E*_m_ = 2 and 5, respectively, whereas the interphase thickness only reduces mildly from 215 nm for *E*_int_/*E*_m_ = 10 to *t* ≈ 198 nm and 188 nm with increasing interphase modulus to *E*_int_/*E*_m_ = 20 and 50. In this regard, one may note that an order-of-magnitude increase in the modulus of the interphase has already been found in nanocomposites with rubber matrix,^47^ and even larger increases have been deduced based on variations in glass-transition temperature.^48^ The estimated thicknesses of interphase point to the presence of long-range interfacial interactions affecting the relaxation behavior of polymer chains relatively deep into the SF matrix. While a propagation distance of ∼20 nm has been suggested for the polystyrene/SiO_2_ interface with a weak interfacial interaction, interphase propagation distances varying between 60 and 250 nm have been reported for poly­(methyl methacrylate)/SiO_2_.^49–51^ Using an estimate of 3*R*_g_ to 11*R*_g_ for the propagation distance,^50^ *R*_g_ being the radius of gyration, and also the reported values of *R*_g_ between 8 and 16 nm for SF and regenerated SF,^52–54^ one arrives at an estimate of 24–176 nm for the propagation distance. The upper end of the estimated range comes close to the interphase thicknesses required for explaining the observed enhancement in the elastic modulus of the composite based on the Halpin–Tsai theory.

For the SF/ZnO 10 sample, ZnO particles take the shape of rod-like fibers (Figure 2d) with a diameter of ∼30.7 μm and an aspect ratio of *l*/*d* ≈ 10.27. Based on the average and maximum measured values of *E*_m_, respectively *E*_m_ = 204.86 MPa, and 247.73 MPa, the theory predicts *E*_c_ = 250.85 MPa and 303.03 MPa. The fact that the measured *E*_c_ in this case is lower than that for SF/ZnO-P at the same filler content of 10 wt %, i.e. 528.38 MPa as compared to 575.0 MPa, is also consistent with the formation of a stiff interphase in the latter case as a result of particle sizes being on the order of a few hundred nanometers.

For the SF/ZnO 1 sample, globular particles are ∼3–5 μm in size (Figure 2b), with an aspect ratio of ∼1.5. The corresponding volume fraction is 0.24%, and the theory effectively predicts *E*_c_ *≈ E*_m_, i.e. 206.45 MPa and *E*_m_ = 249.65 MPa based on the average and maximum measured values of *E*_m_. The second value is compatible, within the errors, with the experimental value of 285.66 MPa for *E*_c_. The SF/ZnO-T specimen contains 10 wt % ZnO-T, with the arm length being 20–30 μm and their tip diameters between 1 and 4 μm. For an arm length of *l ≈* 25 μm, and an average diameter of *d ≈* 2.5 μm, one can estimate an aspect ratio of 10 for individual arms. Even using the largest measured value of *E*_m_ = 247.73 MPa, the theory predicts *E*_c_ = 301.95 MPa, significantly lower than the average measured value of 705.6 MPa. In fact, theoretical predictions come close to the measured value only for an unreasonably large aspect ratio of ∼175. This is suggestive of a strong load-carrying role for the network of interconnected ZnO-T in the matrix. Given the microscale dimensions of the ZnO-T, the discrepancy cannot be explained in terms of the formation of an interphase. However, it is known that effective properties of composites could drastically change beyond a critical concentration of the fibers, also called the percolation threshold, where networks of interconnected fibers begin to emerge and contribute to the overall properties.^55,56^ Hence, the comparison above points to the possibility of a percolation effect. To examine whether the fiber concentration in this case is large enough to cause percolation, we use the excluded volume method to estimate the percolation threshold according to *n*_c_ *V*_exc_ = C, where *n*_c_ = *v*_f_/*V*_p_ is the number of particles per unit volume, *V*_p_ being volume of a single tetrapod, *V*_exc_ the excluded volume calculated for a pair of ZnO-T, and a constant *C*.^57^ Approximating the excluded volume of ZnO-T as *V*_exc_ ≈ 8*πdl* ^2^,^58^ and letting *V*_p_ = *πd* ^2^ *l*, the critical volume fraction is estimated as $\nu_{f}\approx \frac{Cd}{8l}$ . For *C* in the range of 0.8–3,^57^ with *l*/*d* ≈ 10, one estimates the volume fraction threshold to be 1–3.75%. The large effective modulus found for a volume fraction of 2.5% is comparable with the estimated threshold of percolation for ZnO-T. We note that percolation in ZnO-T-silicone composites has already been reported at a 5% volume fraction, in agreement with the current analysis.^18^ To examine whether the contribution of an interconnected network can raise the modulus to the measured level, we highlight that the elastic modulus of a network scaffold can be estimated using the Gibson-Ashby model of open-cell foams as $E_{NW}=C_{1}{\left(\frac{\rho_{NW}}{\rho_{s}}\right)}^{2}E_{s}$ , where *C*_1_ is a geometrical/structural constant of the order of unity, *ρ*_NW_ and *ρ*_s_ are the densities of the network and solid phase, respectively, and *E*_s_ - the modulus of the solid phase.^59^ Since the elastic modulus of our composite is much larger than that of the matrix, it is reasonable to consider *E*_c_ ≈ *E*_NW_. Letting $\frac{\rho_{NW}}{\rho_{s}}\approx v_{f}$ , with *v*_f_ = 0.025, *E*_c_ = 705.6 MPa and *E*_s_ = 130 GPa, one arrives at *C*_1_ = 0.11. This is comparable to, although somewhat lower than, the values reported in the literature for the prefactor *C*_1_,^60,61^ suggesting that intertetrapod connections after percolation are probably key for the superior mechanical behavior demonstrated by ZnO-T composites.

Finally, SF/ZnO-P films show the highest elongation at break along with improved toughness. The large elongation at break of SF/ZnO-P samples compared to both SF/ZnO-T and SF/ZnO 10 samples can be explained in terms of the low aspect ratio, i.e. near-spherical geometry, and nanoscale dimension of the reinforcement particles, which lead to a more homogeneous stress distribution and deformation throughout the matrix, suppressing crack growth and brittle behavior.^62^ In comparison, high-aspect-ratio fibers with dimensions reaching tens of micrometers in both SF/ZnO-T and SF/ZnO 10 can cause high local stress concentrations,^63^ hence promoting brittle fracture. Additionally, as larger interfacial flaws are more expected to form in the presence of larger fillers, it is more likely for larger flaws to preexist in both SF/ZnO-T and SF/ZnO 10, hence inducing brittle behavior in these samples. Although previous studies suggest a mixed effect of nanofillers on the strain-to-failure in polymer composites, it has been noted that nanoparticles increase strain-to-failure in composites with an amorphous polymer matrix,^64^ and that nanofillers can exhibit superior strain-to-failure performance compared to microfillers.^62,65^ The observation that elongation at break first increases with filler content for 0.1 and 1 wt % in SF, i.e. SF/ZnO 0.1 and SF/ZnO 1, and then decreases at the higher filler content of 10 wt % in SF/ZnO 10 suggests the existence of an optimum intermediate filler content that maximizes the strain-at-break,^66^ although in the higher-content samples the shape of the fibers is also expected to have contributed strongly to the reduced elongation, as discussed above.

Thermoplastic-elastomer-based piezoresistive sensors combine traditional elasticity with novel strain-sensing capabilities. Previous approaches for preparation of piezoresistive sensors were based on blending carbon nanotubes or graphene with a thermoplastic matrix.^67–76^ The resistance change ratio (Δ*R*/*R*_0_), where *R*_0_ is the initial resistance before strain is applied and Δ*R* is the difference in resistance relative to *R*_0_, was evaluated. This function of applied strain was compared for SF and SF/ZnO films (Figure 5). The samples were characterized by a linear piezoelectric behavior, indicating that the electrical resistance follows a direct correlation with mechanical deformation, increasing linearly upon tensile loading. The piezoresistive sensitivity of the composites was then quantified by the Gauge Factor (GF), which shows higher values at high strain for the SF/ZnO-T compared to the SF film. The GF variation can be ascribed to the change in their aspect ratio, and further investigation is needed to support this hypothesis. Finally, in view of potential applications of such functional composites as smart sensors, reversibility and response times of the composite have been investigated (Figure S4). The results suggest a response and recovery time of about 200 ms, a value comparable to those of different SF-based composites reported in the literature.^77–79^

**Figure 5:**
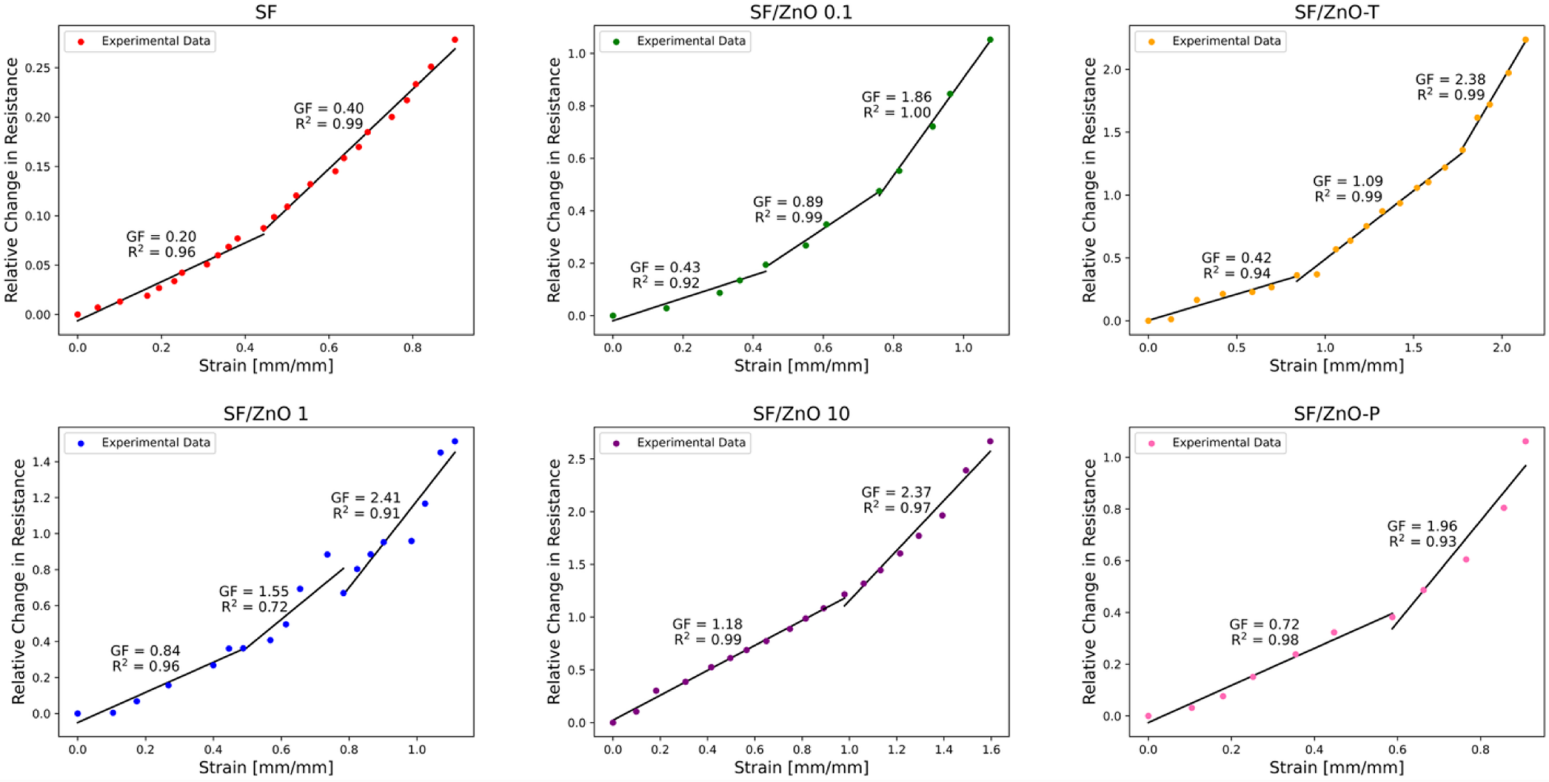
Comparison of resistance change ratio (Δ*R*/*R*_0_) values under strain of SF, SF/ZnO, SF/ZnO-T, and SF/ZnO-P films. Dots represent experimental values, while straight lines indicate the GF values of strain sensors as a function of strain in different regions of the tests.

## 4 Conclusions

In this study, we investigated solvent-assisted evolution of ZnO structures in SF composites as a function of dispersed ZnO-T microparticle content. Globular and microrod-shaped ZnO particles were generated, increasing ZnO-T initial content. We have scrutinized the effect of ZnO microparticles’ branching on the mechanical properties of silk fibroin composites. Films of SF/ZnO-T composition show a significantly greater increase in mechanical properties compared to those with globular and rod-like ZnO microparticles. The theory predicts that the interface between globular- and microrod-shaped microparticles and SF is an important factor in tuning the effective modulus. Branched ZnO-T particles, with their isotropic orientation compared to globular ZnO particles, optimize both these orientations to achieve the largest enhancement of the composite Young’s modulus. By incorporating ZnO microparticles into SF, we developed a stretchable strain sensor that exhibits a significant improvement in fractional change of electrical resistivity and gauge factor, as compared to neat SF films.

## Supplementary Material



## Notes

Figure S1: low-magnification SEM images of the SF, SF/ZNO 0.1, SF/ZnO 1, and SF/ZnO 10 samples. Figure S2: XRD spectra of SF, SF/ZnO 0.1, SF/ZnO 1, SF/ZnO 10, and ZnO-T samples. Figure S3: DSC and TGA measurements of SF, SF/ZnO 10, and ZnO-T samples. Figure S4: reversibility and response time measurements of the SF/ZnO-T sample (PDF)
