# Supplementary material for: Mechanical and Strain-Sensing Responses in Functional ZnO Tetrapod - Silk Fibroin Composites: A Detailed Investigation of the Roles of Filler Size and Shape
Source: ACS Omega. 2026 Jun 18;11(25):36711–22. doi: 10.1021/acsomega.5c13118 (PMC13325081; doi:10.1021/acsomega.5c13118)
Supplement: Supplementary file 1 [file ao5c13118_si_001.pdf]

Supporting Information

Mechanical and strain sensing responses in functional ZnO tetrapods - silk fibroin composites: a detailed investigation on roles of filler size and shape

Rocco Malaspina,[a)] Martina Alunni Cardinali,[b)] Hamed Haftbaradaran,[c)] Danila Maltsev,[d)] Hira Abdullah,[e)] Horst-Günter Rubahn,[e)] Alessandro Di Michele,[a)] Anna Donnadio,[f)] Paola Sassi,[b)] Yogendra Kumar Mishra,[e)] Nicola M. Pugno,[c),g)] Luca Valentini[d)*]

a) *Department of Physics and Geology, University of Perugia, Via A. Pascoli, 06123 Perugia, Italy*

b) *Department of Chemistry, Biology and Biotechnology, University of Perugia, Perugia, Italy*

c) *Mechano-X Labs, Department of Civil, Environmental, and Mechanical Engineering, University of Trento, 38123 Trento, Italy*

d) *Department of Civil and Environmental Engineering, University of Perugia, Via G. Duranti, 06125 Perugia, Italy*

e) *Smart Materials, Mads Clausen Institute, University of Southern Denmark, Alsion 2, DK-6400, Sønderborg, Denmark*

f) Department of Pharmaceutical Science, University of Perugia, Perugia, Italy

g) School of Engineering and Materials Science, Queen Mary University of London, London, E1 4NS, UK

*E-mail: luca.valentini@unipg.it

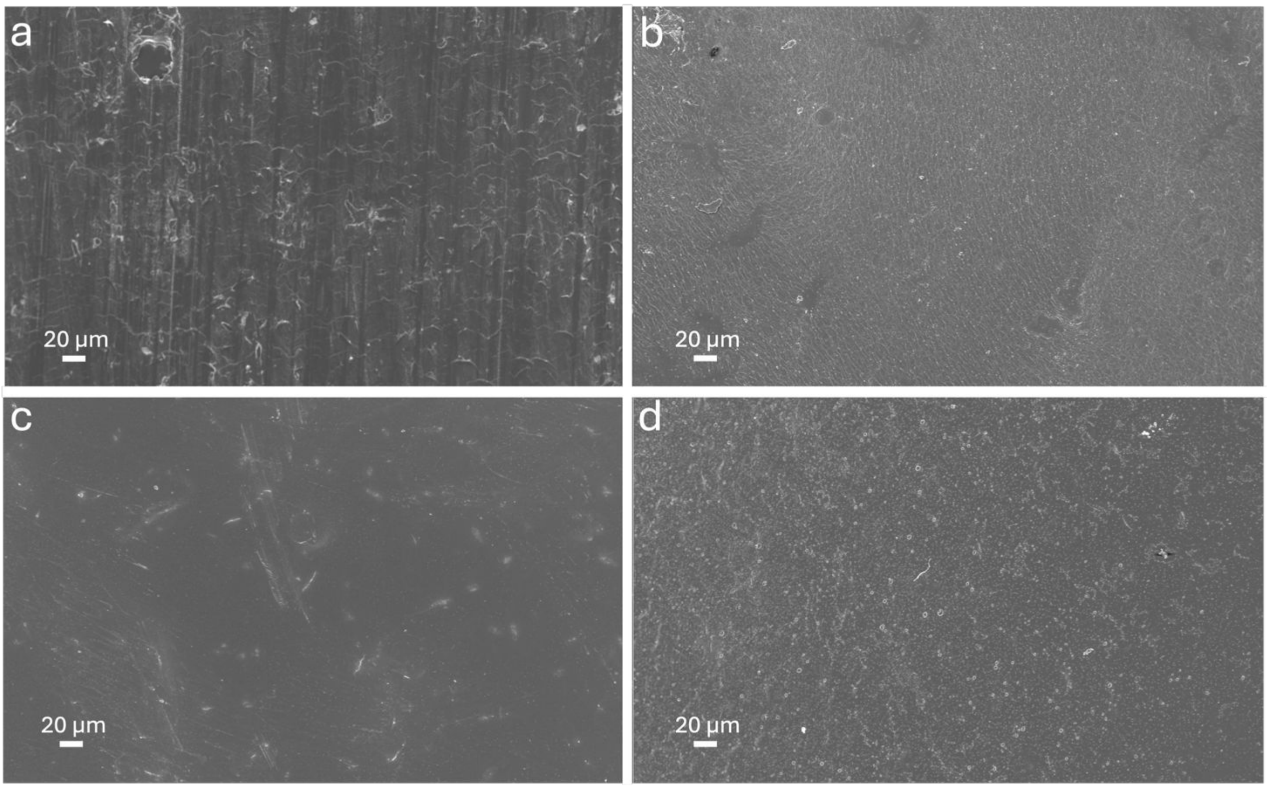

Figure S1. Low-magnification SEM imaging of (a) SF, (b) SF/ZnO 0.1, (c) SF/ZnO 1 and (d) SF/ZnO 10 samples.

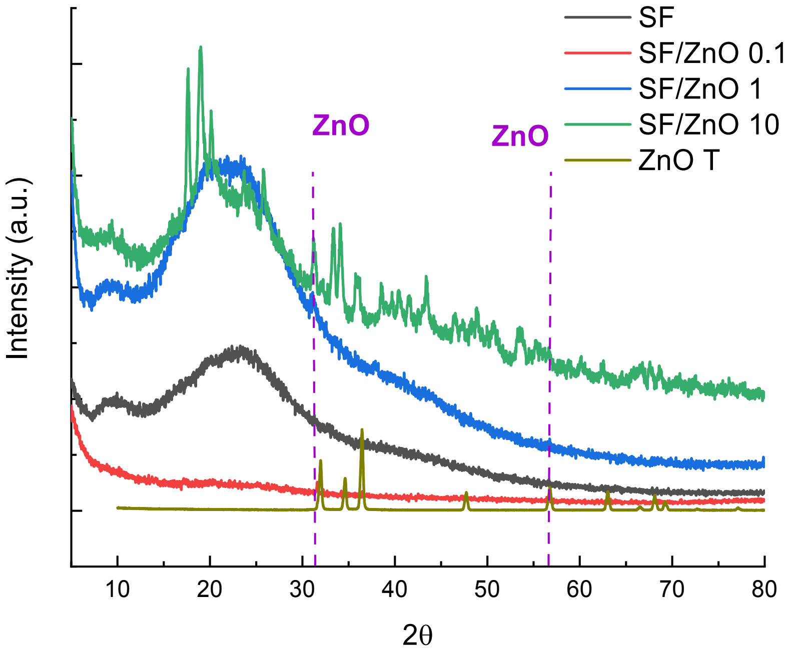

Figure S2. XRD profiles of SF, SF/ZnO 0.1, SF/ZnO 1, SF/ZnO 10 and ZnO-T samples.

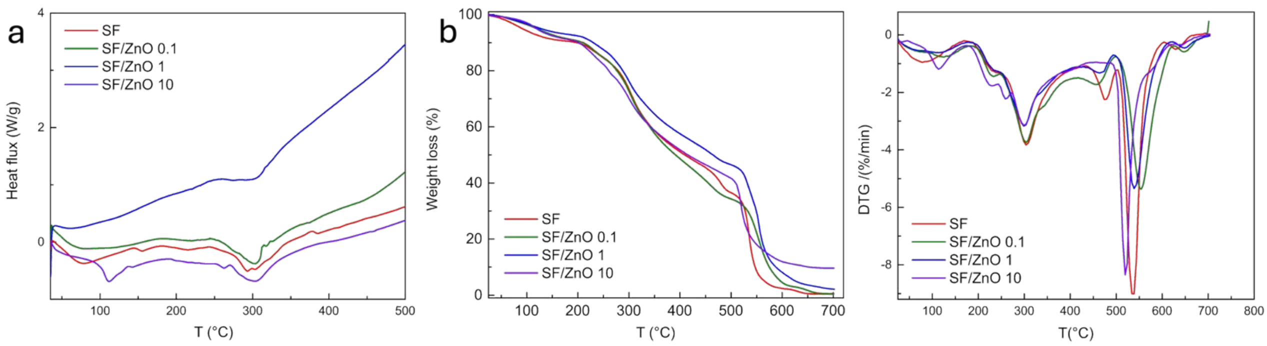

Figure S3. DSC (a) and TGA (b) thermograms of SF, SF/ZnO 0.1, SF/ZnO 1 and SF/ZnO 10 samples. The DSC scans of the samples show a small peak around 50–100 °C that is due to bound water evaporation while TGA analysis shows in the temperature range of 82–150 °C, one step mass loss that it is likely due to zinc formate.[S1,S2]

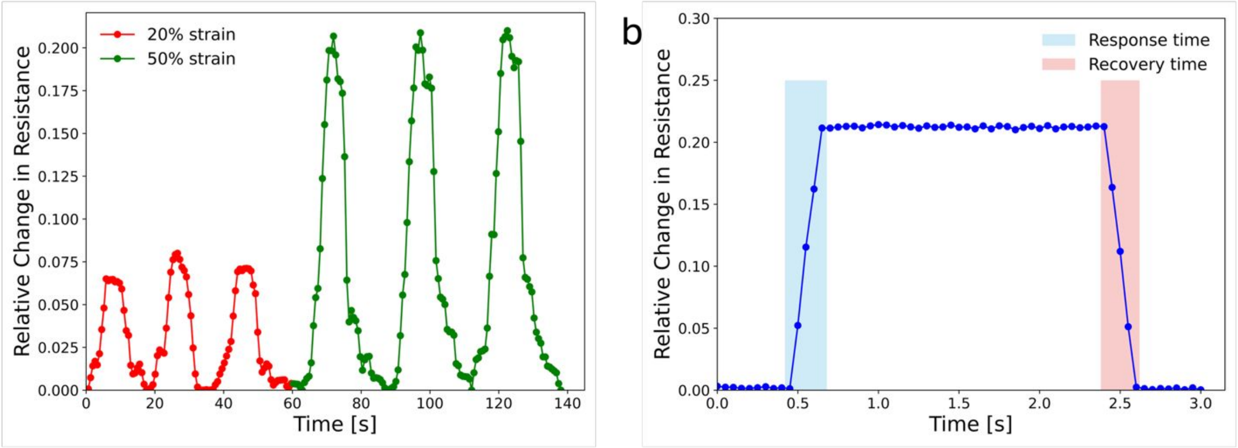

Figure S4. Reversibility at 20% and 50% strain (a) and response and recovery times at 20% strain (b) of the SF/ZnO-T sample.

References

S1. Zhang J, Liu YY, Zeng JL, Xu F, Sun LX, You WS, et al. Thermodynamic properties and thermal stability of the synthetic zinc formate dihydrate. J Therm Anal Calorim. 2008;91(3):861–6.

S2. Wang F, Yu H yang, Gu ZG, Si L, Liu Q chun, Hu X. Impact of calcium chloride concentration on structure and thermal property of Thai silk fibroin films. J Therm Anal Calorim. 2017 Nov 1;130(2):851–9. doi:10.1007/s10973-017-6388-z
